# The Effect of a Points System on Incentivizing Academic Behaviors of Attending Ophthalmologists

**DOI:** 10.3390/healthcare9030340

**Published:** 2021-03-17

**Authors:** Darren A. Chen, Emily Cohen, Gary J. Lelli

**Affiliations:** 1 Weill Cornell Medical College, New York, NY 10021, USA; dac4001@med.cornell.edu (D.A.C.); eyc4002@med.cornell.edu (E.C.); 2 Weill Cornell Medicine Ophthalmology, New York, NY 10021, USA

**Keywords:** ophthalmology, incentives, mentorship, productivity

## Abstract

(1) Background: Little is known regarding the best ways to promote academic throughput within the ranks of attending ophthalmology physicians. The purpose of this project is to evaluate the effect of a monetized points system on incentivizing research output and other academic activity in academic ophthalmology attendings. (2) Methods: This is a retrospective study of 15 academic ophthalmology attendings at a single academic teaching hospital from 1 July 2015 to 30 June 2020. A points system was implemented in the 2017 academic year (1 July 2016–30 June 2017), in which ophthalmology attendings accrued points for eight categories of academic achievement. We compared the overall number of publications, number of first/senior author publications, and corresponding impact factors of journals via the PubMed database in the two years of data before and after the points system was implemented. We analyzed points awarded for eight categories of academic achievement in the first, second, and third year of the program. (3) Results: There was no significant change in research productivity for attending ophthalmologists after institution of the points system. From 2017 to 2019, Mann–Whitney analysis revealed a significant increase in points awarded for mentorship per physician (*p* = 0.013). (4) Conclusions: Our data suggest that within the framework of the points system, attendings—rather than prioritizing publications—gravitated towards mentorship activities to accrue points.

## 1. Introduction

While scholarly activity is required of most ophthalmology residents in academic teaching hospitals, it is not always required of academic attending physicians. Full-time faculty have large time commitments dedicated to patient care, resident management, education, mentorship, and administration. There are specific opportunity costs to carrying out research, especially in a high-pace academic ophthalmology department—namely, a loss of revenue, teaching opportunities, and work-life balance if time is shunted towards research. Other physicians in similar types of sub-surgical specialties, such as urology or otolaryngology, must also find ways to balance meeting surgical volumes and producing research. Considering that academic ophthalmology revolves around three core tenets—patient care, medical education, and research—in order to provide the best patient care and medical education, academic institutions look towards their attending physicians to lead their residents and medical students in projects that aim to push the boundaries of their respective fields. Supporting these research efforts is of paramount importance. 

There have been many studies published on promoting better clinical outcomes through different compensation schemes for physicians [1,2]. For example, one particular randomized clinical trial published in JAMA showed that an increased end-of-year bonus leads to significantly improved quality of care [2]. While financial incentives have often been shown to improve patient care, it is unknown whether or not these incentives would lead to similar improvements in academic productivity. With respect to research output, there has been an abundance of research aimed at the resident-physician level, but the same cannot be said for attendings [3,4,5,6,7,8,9]. A key difference between resident and attending engagement in research should be noted: while residents are often required to reach research quotas for graduation or program requirements, attendings are often free from these quotas. Thus, the same incentive schemes designed to incentivize residents to work harder on research they are already engaged in may not work for attendings who would need to start projects from scratch. 

Bibliometric analyses of ophthalmology publications in the past have shown that on average, ophthalmologists are a prolific group. A study on ophthalmologists associated with the American Academy of Ophthalmology found that 100 random ophthalmologists taken from an ophthalmologic subspecialty from one year will publish approximately 37 articles, with those in vitreoretinal and neuro-ophthalmology publishing the most [10]. Scholarly productivity and associated impact factors of research also increase with fellowship training and advancement in academic rank from assistant professor to professor [11]. In a study on productivity of ophthalmologists in academic departments through the United States, authors found significantly higher research output amongst faculty with higher academic rank [12]. This is unsurprising in the context of the ophthalmology landscape given the current trends towards fellowship training. Ophthalmologists who decide to pursue fellowship training and positions at academic medical centers are the same ones who enjoy research and have the skills to publish meaningful papers and ascend the academic ladder. Depending on their respective cultures, academic institutions also have varying degrees of emphases on research productivity. The main question for institutions with lower than average research productivity then is how to incentivize their faculty to publish more frequently. 

Our team instituted a monetized points system that was designed to increase academic throughput. The purpose of this study is to evaluate the effect of this points system on incentivizing endeavors in areas such as research, mentorship, and administrative responsibilities. 

## 2. Materials and Methods 

The Weill Cornell Medicine institutional review board granted an exemption for this project.

**Figure 1:**
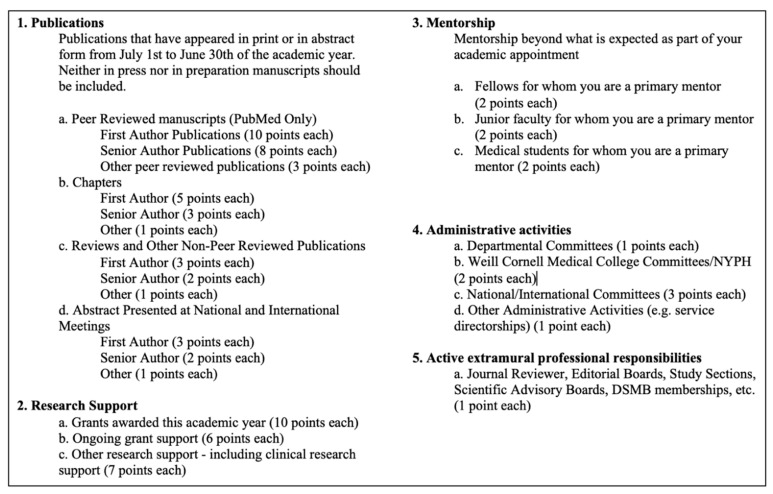
Breakdown of points awarded.

A points system was implemented starting in the 2017 academic year (1 July 2016—30 June 2017), in which ophthalmology attendings were able to accrue points for eight categories of academic achievement (mentorship, publications, research, administrative activities, educational activities, extramural professional activities, honors/awards, and philanthropy)—see Figure 1. Differing amounts of points could be obtained depending on the degree and difficulty of achievement; points were then converted to an end-of-year monetary bonus. For publications, increasing amounts of points were credited depending on the type of publication and the degree of contribution. Points were collected at the end of the academic year and reviewed by members of the practice administration team. 

The incentive plan was initially created by a compensation committee comprised of four faculty members from different ophthalmology subspecialties and the department administrator. The goal of the points system was to incentivize academic achievements that mirror the natural components necessary for academic progression from assistant to associate to full professor. The rollout of the points system was introduced to the faculty by way of a series of information sessions over the course of a year. On multiple occasions, the description sheet of the points system (Figure 1) was given to faculty. Prior to the first year of formal implementation, faculty received a form that allowed them to fill out categories for achievements from the prior year, so that they could obtain an understanding of how the points system would work, how many points they would have hypothetically accrued, and what their bonus would have been for that year. The yearly monetary bonus pool was created from approximately 1% of fee collections from all faculty, which varied depending on the revenue for that year. The faculty would be grouped into four quartiles of possible bonus sizes depending on their academic achievement for the year compared to their peers. Faculty were grouped to receive small, medium, large, or no bonus which corresponded to a monetary award ranging between USD 0, 2000–5000, 5000–10,000, and 15,000–25,000 depending on revenue in the bonus pool for that specific year. 

We retrospectively reviewed the publications of all eligible ophthalmology attendings who qualified for bonuses under the points system. Attendings were eligible for the bonus if they had been a member of the faculty for three years. In total, fifteen ophthalmology attendings qualified, and using the PubMed database, we reviewed all of their publications from academic years (AY) 2015 to 2020. We categorized the publications as manuscripts, reviews, case reports, replies, and other. For each attending, we recorded the overall number of publications, number of first/senior author publications, and corresponding impact factors of journals in the three years of data before the incentivized system was implemented (AY15–AY17) with three years of data afterwards (AY18–AY20) for a total of six years of data. Impact factors of journals were sourced from Clarivate Analytics. Note that we consider the articles published in AY17 to be “before” the implementation of the points system since we are under the assumption that the majority of the work is often completed the year prior to an article appearing in publication. In addition, we also analyzed tracked points awarded to ophthalmologists for the eight categories of academic achievement in the first, second, and third years of the points program (AY17–AY19). 

For statistical analysis, we used Mann–Whitney U Pairwise testing to evaluate differences in publications before and after implementation of the system. Mann–Whitney testing was also used to compare differences in points accrued in the different categories in the years before and after implementation of the system. Note that because AY17 was the first year of data collection for the points program, we used Mann–Whitney U testing, comparing medians of all data available from AY18–19 compared to AY17 for a more robust statistical analysis. All statistical analysis was performed using Microsoft Excel and Prism. All *p*-values calculated were two-tailed, evaluated at the 0.05 alpha level for significance.

## 3. Results

Table 1 shows Mann–Whitney U testing of change in publications, change in number of first/senior authorship, and change in impact factor in the two academic years before and after implementation of the system. There was no significant change in the number or type of publications produced by attendings, the number of first/senior author publications, or impact factor of publications. There was significantly more involvement in mentorship from AY17 to AY18–19, as shown in Table 2. Mean points awarded for mentorship per physician increased from 1.06 in AY17 to 5.67 in AY18/19. Of note, only 3 out of 15 attendings reported points for mentorship during AY17 compared to 11 out of 15 attendings in AY18 and 8 out of 15 in AY19. Overall, the total points awarded for each attending did not significantly change from AY17 to AY18/19. 

## 4. Discussion

Performance-based incentives are commonly used in virtually all industries, medicine being no exception. Compensation systems such as physician pay for performance have revealed that larger bonuses can result in greater adherence to evidence-based care metrics [2]. The idea of employing such a system on academic research may seem counter to the traditional, idealized vision of pursuing knowledge for knowledge’s sake, but it is difficult to ignore the large financial drivers of academic research. 

While our results do not indicate that the points system caused a change in research productivity by attendings, it was associated with an increase in mentorship activity. Similar to any other specialty, the role of mentors in ophthalmology is vital in influencing the career paths of medical students, residents, and fellows [13,14,15]. Previous studies have shown that successful mentorship relationships are characterized by factors such as reciprocity, personal connection, and clear expectations; meanwhile, unsuccessful relationships are characterized by a lack of commitment and personality differences among other factors [16]. Administrative models that incentivize mentorship could therefore be invaluable to promoting a clear set of expectations for mentorship activity while encouraging attending physicians to seek out mentees who share similar personalities and interests. However, caution should be taken, because the clear unintended consequence is that attendings could attempt to complete only the minimum requirements to ensure the reward. 

One question that arises from this increase in mentorship is why did our points system not lead to increases in other categories of academic productivity as well? One likely explanation is that the barrier to entry for mentoring students is the lowest out of all the categories. Compared to the other endeavors, mentorship allows for schedule flexibility that is lacking in the others and represents a straightforward and guaranteed path to accruing points within the system. Furthermore, as in any behavior economic study, establishing causation can be difficult since it is nearly impossible to account for all of the changing dynamics in the environment. The statistically significant correlation found within this study along with the temporal relationship between establishing the points system and increase in mentorship activity over the next two years does suggest that the points system was at the least one factor that promoted mentorship. With the correlative data, we acknowledge that there is the possibility of post hoc ergo propter hoc fallacy. 

Like any other field of academia, faculty are likely motivated to publish for a combination of reasons, including achievement, enjoyment, recognition, rewards, and institutional pressure [3]. The responsibility of balancing these intrinsic and extrinsic motivators falls into the purview of department heads and administrators. Most studies that have aimed to increase research productivity in medicine have targeted residents rather than attending physicians [4,5,6]. One model that has effectively increased neurology resident research activity is a compulsory system that requires graduating residents to complete a peer reviewed publication or presentation [6]. A consequence of this system is that faculty involvement in research also saw an increase since residents need mentors to help them complete their projects. Recently, two systematic reviews of interventions to increase research publications in medical residents concluded that factors such as departmental leadership and curricula are associated with increased research efforts [7,8]. Whether such factors would lead to similar increases in research output for attending physicians is unclear, and it may be the case that any type of research-stimulating interventions could be subject to a longer time course before there arises any significant benefit.

With respect to incentivized programs geared towards increasing research productivity, there is one particular study that examined the effect of a monetary rewards system on research output among otolaryngology residents [9]. Similar to our incentivized model, residents accrued different number of points for the type of research articles produced, progressive steps in the research process, presentations, and impact factors. Points were then converted yearly to a monetary bonus that residents could use for educational expenses. Data were compared from 1998 to 2011, with 2004 being the year the rewards system was implemented. Results showed a statistically significant increase in mean publication output per resident per year from 0.13 (95% CI, 0.03–0.23) before the rewards system to 0.43 (95% CI, 0.26–0.60) after the rewards system (*p* = 0.004). Approved institutional review board projects increased from 0.47 (95% CI, 0.18–0.75) per resident per year to 1.29 (95% CI, 0.96–1.63) (*p* = 0.007). However, as demonstrated by our study herein, such a rewards system fails to incentivize attending physicians in the same way. One can speculate on the reasons for this, but the discrepancies in professional responsibilities between attending physicians and residents coupled with the results shown in this paper suggest that there is a fundamental difference in motivating factors that influence research activity between these two groups. Another study on surgery faculty members at Baylor College of Medicine evaluated a similar type of points system [17]. In this study, faculty self-reported their activities in five major academic categories—education/mentorship, innovation, academic service, peer review, and research; they were subsequently grouped into three tiers—top 10%, top third, and top half—depending on their achievements and received bonuses in accordance to their productivity. Implementation of this system led to significant increases in presentations and publications, with the caveat being that there were several confounders, including a concomitant department push towards recruiting new faculty with established research experience, expanding research support teams, and creating additional funding/grant opportunities. In the context of these studies, it is important to realize that incentive systems are institution specific; certainly, there are trends and overarching themes such as points systems that undoubtedly motivate faculty towards academic activity, but these systems must be tailored to the institution’s personnel and culture.

The increase in mentorship activity seen in our results suggest that at least some physician behavior was corelated with implementation of the points system. Mentorship is especially important for ophthalmology residents who have the option of pursuing fellowship or going directly into a comprehensive ophthalmology practice. While most graduating ophthalmology residents are comfortable with their clinical practice skills, it has been reported in the literature that up to 60% did not feel well prepared in the non-clinical aspects of managing a practice [18]. It is not unreasonable that residents would expect some sort of training in these areas of practice management, and this would be one area in which further efforts toward promoting mentorship could better help prepare graduating residents for the transition to full-time clinical practice. 

The limitations of our studies are as follows: First, while the single departmental/institutional nature limits the generalizability of the study, it should be noted that each institution incentivizes and compensates physician behavior differently. In this study, we demonstrate one particular academic compensation scheme that was shown to improve mentorship activities. This particular limitation also represents the pragmatic nature of such a study, because all institutions implementing their own academic compensation programs will experience similar challenges in generalizability. Traditionally, physician compensation studies have also been performed at single institutions [2,3,4,5,6,9,19]. Second, three years of follow-up may or may not represent a long enough time course to see an uptick in publications. However, given the nature of clinical research in ophthalmology, it would be rare for a study to take longer than 3 years from conception to manuscript publication. A fourth year of data post implementation would have been included, but the points system was halted for AY20 due to financial constraints from the coronavirus pandemic. Third, at least part of the apparent increase in mentoring activities may be due to more diligent documentation by attendings. 

Based on our retrospective analysis, mentorship is one particular area that would likely be bolstered by monetary incentives and could greatly benefit medical trainees. Departments should recognize though that any incentivized mentorship programs need to have strict guidelines for what constitutes mentorship in order to deter physicians from exploiting the system for rewards. Nevertheless, for institutions without formalized mentorship programs for fellows, residents, or medical students, such a system would be instrumental in influencing career trajectories. More time is likely needed to determine if research productivity in ophthalmology can be increased through such a system described herein. Point systems and monetary awards can and have been shown in the past to positively drive research productivity [4,6,7,9,19,20,21]. Iterative changes to incentivized systems over time at single institutions are a potential starting point for quality improvement projects. If an institution’s overarching goal is to encourage more publications, incentivized points systems will need to be carefully tailored to the culture and personnel of the specific institution. 

## Figures and Tables

**Table 1:** Mann–Whitney U pairwise testing of median publications before and after implementation of points system.

	AY15–17	AY18–20	*p* Value
Manuscripts	63	67	0.610
Reviews	20	14	0.267
Case Report	20	19	0.719
Reply	6	3	0.624
Other	3	7	0.617
First/senior author	35	34	0.522
Median Impact factor	3.09	3.08	0.689

**Table 2:** Total average points awarded in AY17 compared to AY18–19. Mann–Whitney U pairwise testing of points awarded before and after implementation of monetary bonus.

	AY17	AY18–19	*p* Value
Mentorship	19	102	0.013
Publications	500	357	0.682
Research	152	160	0.827
Admin	183	218	0.842
Education	425	533	0.928
Extramural	58	70	0.338
Honors/Awards	26	22	0.569
Philanthropy	17	24	0.976
Other	2	4	0.764
Total	1382	1429	0.803

## Data Availability

The data presented in the study are available by request due to privacy.
